# Interweaving Elastic and Hydrogen Bond‐Forming Polymers into Highly Tough and Stress‐Relaxable Binders for High‐Performance Silicon Anode in Lithium‐Ion Batteries

**DOI:** 10.1002/advs.202302027

**Published:** 2023-09-26

**Authors:** Daun Jeong, Jinsol Yook, Da‐Sol Kwon, Jimin Shim, Jong‐Chan Lee

**Affiliations:** ^1^ Energy Storage Research Center Korea Institute of Science and Technology (KIST) 14 Gil 5 Hwarang‐ro, Seongbuk‐gu Seoul 02792 Republic of Korea; ^2^ School of Chemical and Biological Engineering and Institute of Chemical Processes Seoul National University 1, Gwanak‐ro, Gwanak‐gu Seoul 08826 Republic of Korea; ^3^ Department of Chemical and Biological Engineering Korea University 145, Anam‐ro, Seongbuk‐gu Seoul 02841 Republic of Korea

**Keywords:** binders, elastic polymers, lithium‐ion batteries, silicon anodes, stress relaxation

## Abstract

A central challenge in practically using high‐capacity silicon (Si) as anode materials for lithium‐ion batteries is alleviating significant volume change of Si during cycling. One key to resolving the failure issues of Si is exploiting carefully designed polymer binders exhibiting mechanical robustness to retain the structural integrity of Si electrodes, while concurrently displaying elasticity and toughness to effectively dissipate external stresses exerted by the volume changes of Si. Herein, a highly elastic and tough polymer binder is proposed by interweaving polyacrylic acid (PAA) with poly(urea‐urethane) (PUU) elastomer for Si anodes. By systematically tuning molecular parameters, including molecular weights of hard/soft segments and structures of hard segment components, it is demonstrated that the mechanical properties of polymer binders, such as elasticity, toughness, and stress relaxation ability, strongly affect the cycling performance of Si electrodes. This study provides new insight into the rational design of polymer binders capable of accommodating the volume changes of Si, primarily by judicious modulation of the mechanical properties of polymer binders.

## Graphical abstract

To address the volume change issues of Si anodes for lithium‐ion batteries, highly tough polymer binders exhibiting mechanical robustness, elasticity, and stress‐relaxing ability are developed by interweaving poly(acrylic acid) (PAA) and poly(urea‐urethane) (PUU) elastomer. Systematic modulation of various molecular parameters of PUU elastomer enables accessing optimized mechanical properties of binders, thereby achieving long‐lasting Si anodes.

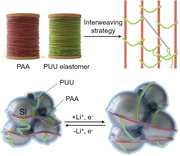

## 1 Introduction

As lithium‐ion batteries have powered a revolutionary transition to sustainable energy generation over the past three decades, tremendous progress has been made in developing advanced electrode materials that deliver high energy densities with a long lifespan.^[^ ^1^ ^]^ Si has been broadly recognized as one of the promising anode materials for lithium‐ion batteries due to its high theoretical specific capacity (3590–4200 mAh g^−1^ for Li_15_Si_4_–Li_22_Si_5_), low operating potential (0.4 V vs Li/Li^+^), and natural abundance.^[^ ^2^ ^]^ However, Si anodes inevitably experience enormous volume changes of over 300% upon Li^+^ insertion and extraction during cycling, hindering their practical utility.^[^ ^2^, ^3^ ^]^ The repetitive volume expansion and contraction of Si loosen the particle‐to‐particle contact between the Si and conductive carbon networks, resulting in the electrical isolation of Si active materials and electrode delamination.^[^ ^4^ ^]^ Furthermore, the considerable volume changes induce the pulverization of Si particles which is accompanied by fracture and reconstruction of the solid electrolyte interphase (SEI) layer, potentially causing electrolyte consumption and capacity fading.^[^ ^5^ ^]^

Extensive efforts have been made to address the aforementioned failure mechanisms of Si anodes, including nanostructuring of Si,^[^ ^6^ ^]^ introductions of silicon oxides (SiO*_x_*, *x*≈1),^[^ ^7^ ^]^ composites with carbonaceous materials,^[^ ^8^ ^]^ and electrolyte additive engineering.^[^ ^9^ ^]^ Besides engineering the Si active materials or electrolytes, designing polymer binders with desired physicochemical properties allows the Si particles to tolerate any external compressive or shear stresses upon cycling.^[^ ^10^ ^]^ PAA is the most established binder for the pure Si electrodes, which primarily relies on the abundant polar carboxylic groups enabling strong hydrogen bonding with the Si surface and providing the appropriate level of viscosities of electrode slurry.^[^ ^11^ ^]^ Despite the advantageous features, PAA cannot sustain the structural integrity of Si electrodes, because the intrinsically stiff PAA backbone with a linear structure is inevitably susceptible to efficient plastic deformation upon the volume changes of Si.^[^ ^12^ ^]^ In an effort to overcome the drawbacks of such synthetic linear polymers, advanced polymer binders adaptive to external stress have been devised, including biomass‐derived branched polymeric binders,^[^ ^13^ ^]^ 3D network cross‐linked binders,^[^ ^14^ ^]^ supramolecular polymeric binders,^[^ ^15^ ^]^ and self‐healable binders.^[^ ^16^ ^]^

As the pioneering studies have aided in shaping the preliminary understanding of the indispensable roles of binders, research communities have reached a tentative consensus that the mechanical properties of polymer binders are essentially the primary factors governing the cycling behavior of Si.^[^ ^10^, ^15^ ^]^ However, there remains an important open question of which mechanical properties of polymers are desirable to alleviate the failure issues of Si. This is perhaps not surprising, in part because building a suitable model polymer system by embracing the multitude of parameters is experimentally challenging.

Herein, we have prepared a mechanically robust and highly elastic PUU elastomer model system that offers control over the molecular weights of the hard/soft segments and their chemical structures. One key to simultaneously accessing mechanically orthogonal properties, i.e., rigidity and elasticity, is to interweave PAA with the PUU elastomer by thermal cross‐linking, thereby leading to a rigid but also highly elastic structure. Consequently, the rigid and stiff PAA domain is responsible for mechanical robustness that retains the overall structural integrity of Si electrodes, while the elastic PUU domain imparts soft and elastic features to effectively relieve the mechanical stresses generated by the volume changes of Si.

## 2 Results and Discussion

**Figure 1:**
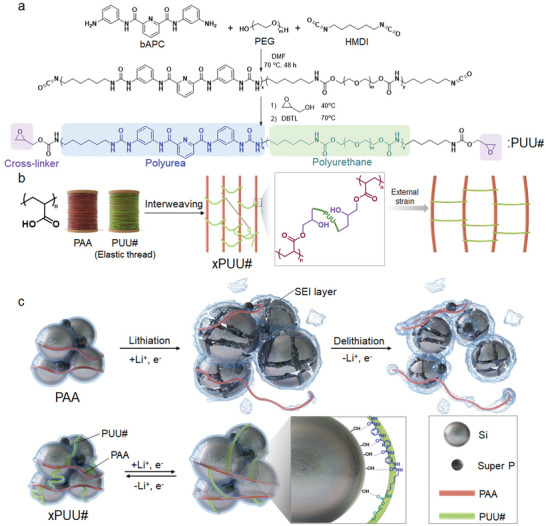
Synthesis of PUU# elastomers and design concept of xPUU# binder system. a) Synthetic route to poly(urea‐urethane) (PUU#), where # indicates the molecular weight of PEG. b) Schematics of preparing an interwoven network structure of xPUU#. c) Proposed working principles of the neat PAA and xPUU# binder systems during cycling.

As a model system of cross‐linkable epoxy‐telechelic PUU elastomers, a series of PUU# was synthesized using *N* ^2^, *N* ^6^–bis(3–aminophenyl)pyridine–2,6–dicarboxamide (bAPC) as the hard segment component, poly(ethylene glycol) (PEG) as the soft segment component, and the terminal epoxides as the cross‐linkable moieties as presented in **Figure** **1**a. Note that the number # in the PUU# indicates the molecular weight of PEG ranging from 400 to 6000 g mol^−1^. Details of molecular characteristics of the PUU#s, including ^1^H and ^13^C NMR spectroscopic and GPC characterizations, are presented in Figures S1–S3 and Tables S1 and S2 in the Supporting Information. Note that across the series of PUU#s, the molecular weights are nearly close in the range of 37–40 kg mol^–1^ (Table S1, Supporting Information). As schematically depicted in Figure 1b, as‐prepared PUU# was employed as a cross‐linker for PAA to construct a polymeric network structure (referred to as xPUU#), wherein the telechelic epoxy groups of the PUU# react with the carboxylic acid moieties of PAA at 120 °C (see Figure S4, Supporting Information). By interweaving the rigid PAA and highly elastic PUU# thread into an integrated network system, orthogonal mechanical features derived from PAA and PUU can be achieved simultaneously. Thus, upon being exerted by external stress, the relatively stiff and rigid PAA domain provides mechanical robustness that maintains the overall structural integrity, while the soft and elastic PUU effectively relieves the stress. In this manner, xPUU# can effectively dissipate the mechanical stress that is generated by the significant volume changes of Si during electrochemical cycling. As illustrated in Figure 1c, the neat PAA binder partially loses physical contact either with Si or conductive carbon (i.e., super P) as Si particles experience volume changes during lithiation/delithiation cycles, which is concomitantly accompanied by pulverization of Si particles and SEI layer.^[^ ^17^ ^]^ Because the linear PAA interacts with Si particles primarily by the one‐dimensional contacts via the carboxylic moieties along the rigid backbone, electrode integrity would be permanently damaged once the applied stress exceeds the critical limit of plastic deformation of PAA.^[^ ^10^, ^12^ ^]^ Interweaving PAA with the PUU brings a sharp contrast, wherein the highly elastic PUU chains anchored on PAA effectively accommodate the stress, which is being applied by lithiation, through chain stretching. Furthermore, particle‐to‐particle contact between the Si and conductive carbon networks can also be retained upon delithiation, primarily attributable to hydrogen bonding between the urea/urethane moieties of PUU and hydrolyzed Si surface,^[^ ^18^ ^]^ as discussed later.

**Figure 2:**
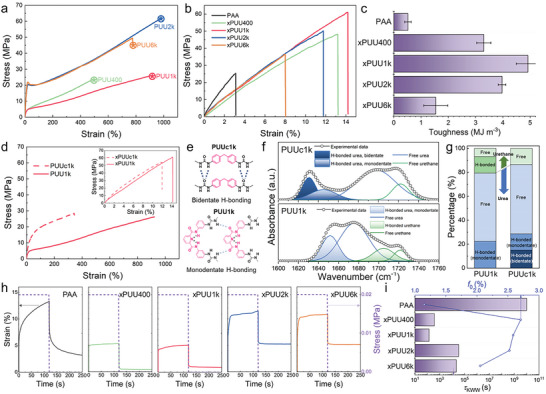
Mechanical properties of the PUU# and xPUU# series. Stress–strain curves of a) PUU#s and b) xPUU#s compared to PAA. Fracture points are denoted by ★. c) A summary of the toughness values of PAA and xPUU#s. d) Stress–strain curves of PUUc1k and PUU1k (inset: xPUUc1k and xPUU1k). e) Proposed hydrogen (H)‐bonded state of PUUc1k and PUU1k in the hard segment. f) Deconvoluted FT‐IR spectra of PUUc1k and PUU1k displaying H‐bonded and free C═O stretching bands. g) Percentages of H‐bonded and free urea/urethane moieties of PUU1k and PUUc1k. h) Creep‐recovery curves of PAA and xPUU#s. i) Relaxation time (*τ*_KWW_) and free volume (*f*_0_) of PAA and xPUU#s at 30 °C, derived from Kohlrausch‐Williams‐Watts (KWW) and Williams‐Landel‐Ferry (WLF) function, respectively.

**Figure** **2** summarizes the mechanical properties of the PUU# and xPUU# series. Note that each xPUU# contains 90 wt.% of PAA and 10 wt.% of PUU#, wherein the carboxylic acid moieties of PAA are partially cross‐linked with the telechelic epoxy groups of PUU#. For the measurements, we prepared the xPUU# samples by solution casting and the subsequent vacuum drying, followed by compression molding and heating at 120 °C to induce cross‐linking. As shown in the stress–strain curves of the PUU# series (Figure 2a), PUU#s exhibit distinct tensile behaviors depending on the molecular weights of the soft segment, #, under a constant strain rate deformation. Both PUU400 and PUU1k showed highly elastic and homogeneous deformation behavior with the average strains at break of 446 and 930%, respectively. However, as the # increases beyond 1k, PUU2k and PUU6k exhibit prominent yield points followed by strain softening. It is also notable that the elongation at break substantially decreases as the # increases from 2k to 6k. This phenomenon is presumably associated with the increased phase separation between the hard and soft segments with increasing the PEG molecular weight,^[^ ^19^ ^]^ which imparts the strain gradient at the hard‐soft domain interface under stress.^[^ ^20^ ^]^ Furthermore, the pronounced phase separation leads to a significant increase in tensile strength values of PUU2k and PUU6k compared with PUU400 and PUU1k, potentially due to stronger hydrogen bonding in the larger hard domain (see Figure S5, Supporting Information), while the unrecoverable deformation with strain softening brings the less elastic features compared with PUU1k.^[^ ^21^ ^]^ Upon being cross‐linked with PAA (see Figure 2b), all the xPUU#s showed greatly improved elongation and tensile strength compared to the neat PAA, primarily attributable to the elastic feature of the PUU#. Interestingly, xPUU1k prepared with the most elastic PUU1k among the PUU# series showed the largest elongation at break of 15%, with a toughness of 4.9 MJ m^−3^, which is almost ten times higher than that of the neat PAA as summarized in Figure 2c. This finding illustrates that the cross‐linked xPUU# reflects the original mechanical characteristics of the PUU# elastomer.

To investigate the effect of the structure of the hard segment component on mechanical properties, we used 4,4–methylenedianiline as a diamine counterpart for bAPC, and the resulting PUU is referred to as PUUc1k (see Figure S6, Supporting Information). Interestingly, there was a striking difference in the tensile behavior of PUU1k and PUUc1k, as shown in Figure 2d. PUU1k exhibited almost three times larger elongation at break (930%) than PUUc1k (346%), which is also observed from their PAA‐cross‐linked xPUU samples as shown in Figure 2d inset, where the difference in the elongation behavior was less pronounced in the xPUU system presumably due to the presence of rigid PAA. We speculate that this intriguing difference in elasticity is associated with different intermolecular interactions in the hard domain. As depicted in Figure 2e, 4,4–methylenedianiline with a symmetric structure enables facile *π*–*π* stacking and hydrogen bonding,^[^ ^22^ ^]^ while bAPC with a bulky aromatic structure makes a relatively loosely‐packed hard domain in PUU.^[^ ^23^ ^]^ As evidenced by deconvoluted FT‐IR spectra displaying hydrogen (H)‐bonded and free C═O moieties of urea and urethane (Figure 2f), PUUc1k exhibited both monodentate and bidentate H‐bonded urea regime, while PUU1k only showed monodentate H‐bonded IR band. As summarized in Figure 2g, PUUc1k possesses a larger amount of H‐bonded hard domain than PUU1k counterpart, which is essentially associated with stronger intra‐/intermolecular interactions due to the closely packed hard segments. Consequently, PUU1k, incorporating bAPC as a hard segment component, demonstrated superior elasticity compared to PUUc1k, leading to the high toughness of xPUU1k under strain deformation.^[^ ^24^ ^]^ This result highlights that the structure of the hard segment component of the PUU plays a dominant role in dictating the tensile properties.

Besides the tensile behavior, it is desirable that the Si binder promptly recovers from the mechanical stresses exerted by the volume changes of Si during the repeated cycling.^[^ ^25^ ^]^ To examine the stress‐relaxing ability of the xPUU# binders, we performed the creep‐recovery experiment as presented in Figure 2h. Upon being applied by a constant stress of 20 kPa for the initial 120 s in the creep zone, PAA showed a gradual increase in the strain value due to its viscoelastic nature, while xPUU#s exhibited an instant increase in the strain as indicative of their elastic feature.^[^ ^26^ ^]^ Upon removing the stress, the strain was released with time, where the strain recovery ratio for a duration of 130 s was 76% for PAA and 92, 83, 54, and 53% for xPUU400, xPUU1k, xPUU2k, and xPUU6k, respectively. It is interesting to note that the xPUU400 and xPUU1k enable efficient stress relaxation compared to PAA, primarily due to their highly elastic features. Although the xPUU2k and xPUU6k exhibited a significant portion of the retained stress, associated with permanent deformation due to the increased phase separation, they also showed much faster stress relaxation behavior than the neat PAA. The exceptional stress‐relaxing ability of the xPUU#s compared to PAA was further corroborated by determining the relaxation time (*τ*) and free volume fraction (*f*_0_) by dynamic mechanical analysis.^[^ ^27^ ^]^ Figure S7 (Supporting Information) shows the master curves of storage moduli obtained using the time‐temperature superposition principle and the Williams‐Landel‐Ferry (WLF) equation, wherein the relaxation time (*τ*) was derived by fitting the master curves with the Kohlrausch–Williams–Watts (KWW) function. As summarized in Figure 2i and Table S3 (Supporting Information), the *τ*_KWW_ values of the xPUU#s were substantially smaller than that of the PAA, and the xPUU400 and xPUU1k exhibited significantly shorter relaxation time compared with xPUU2k and xPUU6k by two orders of magnitude, which is consistent with the stress‐relaxation behavior glimpsed by the creep‐recovery experiment. We attribute the superior stress relaxation behavior of the xPUU#s compared to the neat PAA to the increased free volume formulated by cross‐linking, which in turn restrains the intermolecular interaction with the carboxylic moieties of PAA.^[^ ^27^, ^28^ ^]^ These results indicate that the molecular design of the xPUU# associated with the interweaving strategy essentially provides a remarkable influence over the stress relaxation capability.

**Figure 3:**
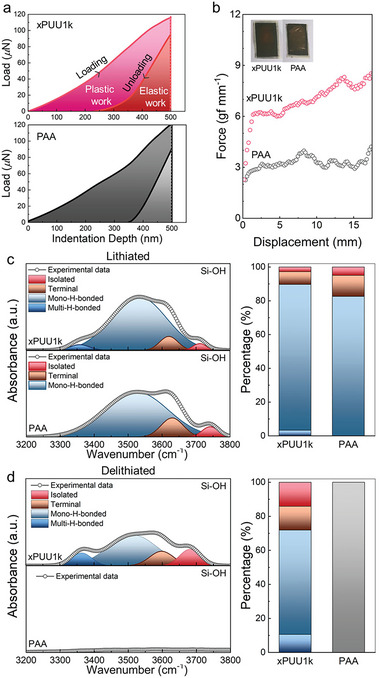
Mechanical properties and H‐bonded states of Si electrodes. a) Nano‐indentation results and b) 180° peeling test profiles of the Si electrodes with PAA and xPUU1k binder. Deconvoluted FT‐IR spectra of the PAA and xPUU1k electrodes after the first c) lithiation and d) delithiation.

Following the previous discussions on the mechanical properties of the xPUU#s, we have prepared a Si electrode by adopting the xPUU1k as a representative binder, which exhibited the highest toughness with excellent stress relaxation behavior across the xPUU# series. Note that the Si electrode with the xPUU1k was prepared by in‐situ thermal cross‐linking of electrode slurry containing Si, super P, PAA, and PUU1k with a weight ratio of 60:20:18:2. **Figure** **3**a shows the representative loading‐unloading curves of the Si electrodes with PAA and xPUU1k obtained by nano‐indentation, where the maximum penetration depth was fixed as ≈2% of the electrode thickness, i.e., 500 nm. The elastic recovery behavior of each electrode was quantitatively assessed by the elastic recovery ratio, which is defined as the fraction of the restored distance upon unloading over the maximum penetration depth.^[^ ^29^ ^]^ It was found that the xPUU1k electrode exhibited a higher elastic recovery ratio of 0.54 than the PAA electrode (0.30), demonstrating the effect of interweaving PAA with the highly elastic PUU1k. The electrode elasticity was also evaluated by comparing the plastic and elastic works obtained by integrating the areas underneath the loading‐unloading curves.^[^ ^30^ ^]^ The ratio between the elastic and plastic works detected from the xPUU1k electrode was 0.47, significantly higher than that of the PAA electrode (0.28), which is consistent with the trend discovered in the elastic recovery ratio of the electrodes.

Since the primary role of the binder is to impart strong interlayer adhesion in the electrode, the adhesive force was intuitively evaluated by the 180° peeling test. As shown in Figure 3b, the xPUU1k electrode exhibited significantly higher adhesion force (≈7.5 gf mm^−1^ on average) than the PAA electrode (≈3.0 gf mm^−1^). This result is consistent with the optical images obtained after the peeling test, in that a relatively larger portion of copper foil was revealed in the case of the PAA electrode compared to the xPUU1k electrode (see Figure 3b inset). One primary factor governing the adhesion force is the degree of intermolecular interactions enabling facile contact between the electrode components. To further expose the underlying molecular‐level origin of the superior adhesive property of the xPUU1k electrode, we have quantified the degree of hydrogen bonding between the binder and the surface silanol moieties of Si by deconvoluting the FT‐IR spectra of the Si electrodes after one cycle of lithiation and delithiation. We have particularly focused on the silanol (Si─OH) regime by assuming that the Si particles are covered with native and partially hydrolyzed SiO_2_ layer so that any changes in the hydrogen‐bonded state of the silanol moieties would reflect the changes in interactions between the Si particles and binder. As presented in Figure 3c,d, FT‐IR spectra of the PAA and xPUU1k electrodes were deconvoluted into four peaks associated with the isolated, terminal, mono‐H‐bonded, and multi‐H‐bonded silanol. Note that the terminal silanol indicates the silanol whose oxygen atoms are H‐bonded only with the hydroxyl groups right next to them.^[^ ^31^ ^]^ As shown in Figure 3c, the fraction of H‐bonded silanol existing in the xPUU1k electrode (90%) is higher than that of the PAA electrode (83%) after lithiation, because the interwoven network structure of xPUU1k maintains the structural integrity of the electrode upon lithiation by enabling facile hydrogen bonding between the urea/urethane moieties and the silanol. Furthermore, it is intriguing to note that the multi‐H‐bonded absorption band is exclusively detected from the xPUU1k electrode, thus demonstrating that xPUU1k facilitates additional hydrogen bonding, which could be evidence of superior adhesive strength as seen in Figure 3b. Strikingly, as shown in Figure 3d, 72% of silanol in the xPUU1k electrode was still H‐bonded even after delithiation, indicating that intimate interactions between the Si particles and the xPUU1k binder are retained well during the volume contraction of Si particles. We speculate that the highly elastic, tough, and stress‐relaxing features of the xPUU1k primarily contribute to reversibly maintaining hydrogen bonding under such an enormous volume‐changing environment. In sharp contrast, none of the silanol absorption bands was detected from the PAA electrode after delithiation, presumably associated with forming the thick SEI layer blocking the silanol signals,^[^ ^32^ ^]^ which has been described later.

**Figure 4:**
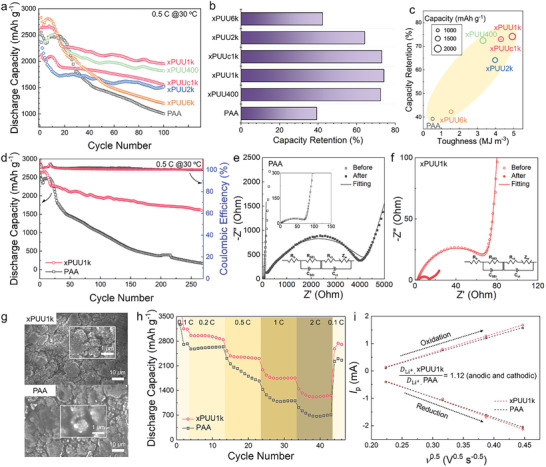
Cycling performance of the Si electrodes. a) Long‐term cycling performance of Li/Celgard2320/Si cells cycled under 0.5 C at 30 °C, where the binder is PAA or xPUU#. b) Capacity retention values of each cell. c) Relationship between specific capacity, capacity retention, and toughness of binders. d) Long‐term cycling performance of the cells with PAA and xPUU1k binder cycled under 0.5 C at 30 °C. Nyquist plot and the equivalent circuit of the cells with e) PAA and f) xPUU1k binder before and after 270 cycles. g) SEM images of the cycled Si electrodes with PAA and xPUU1k binder after 270 cycles. h) Rate capability of the cells cycled under various C‐rates at 30 °C. i) Linear plot of peak current (*I*_p_) versus square root of potential scanning rate (*ν* ^0.5^).

The cycling performance of Li/Celgard2320/Si cells with the series of xPUU# binders was evaluated by comparing it with that of the neat PAA (**Figure** **4**a). Note that the optimized PUU# content in each xPUU# was determined as 10 wt.% as shown in Figure S8 (Supporting Information). It was found that the cells with xPUU# binders delivered relatively higher specific capacities than the cell with PAA. Furthermore, the xPUU1k cell exhibited significantly higher capacity compared to the cell with the xPUUc1k counterpart. It is intriguing to note that the capacity retention values over 100 cycles of the cells (Figure 4b) are reminiscent of the toughness values of xPUU#s, as previously shown in Figure 2c. Indeed, there is a strong linear relationship between the toughness of the binders and cycling performance, including specific capacity and capacity retention, as plotted in Figure 4c. However, when comparing xPUU400 and xPUU2k, xPUU400 exhibited a higher specific capacity compared to xPUU2k, despite its lower toughness. This disparity can be attributed to the more elastic feature of PUU400, which allows for effective stress relaxation, as demonstrated by creep‐recovery and time‐temperature superposition tests. Nonetheless, it should be noted that in general, both specific capacity and capacity retention values tend to increase as the binder toughness increases. This finding shed light on a new insight that the toughness of the binder is a key parameter dictating the cycling performance of Si electrodes. Among the series of xPUU#s, the xPUU1k with the highest toughness exhibits the highest specific capacity and capacity retention of up to 74%, remarkably surpassing that of the cell with PAA (39%). However, the cell with only PUU1k binder, without PAA, did not generate any discharge capacity (see Figure S9, Supporting Information), indicating that stable cycling performance is not guaranteed without the rigid PAA, which maintains the overall structural integrity of the electrode against the large volume change of Si during cycling.^[^ ^33^, ^34^ ^]^

We have further evaluated the long‐term cycling performance of the cell with xPUU1k binder by comparing it with that of the cell with the neat PAA (Figure 4d). The xPUU1k cell showed remarkably stable cycling performance over 270 cycles with 61% of capacity retention, while the PAA cell exhibited an abrupt capacity loss with 7% of capacity retention. See the voltage‐capacity profiles of each cell in Figure S10 (Supporting Information). Furthermore, as shown in the EIS spectra in Figure 4e,f, the xPUU1k cell exhibited a significant decrease in the charge transfer resistance (*R*_ct_) from 66 to 14 Ω upon cycling, whereas the PAA cell showed a dramatic increase in the *R*_ct_ from 68 to 4017 Ω. This result indicates that the xPUU1k binder promotes stable SEI layer construction compared to the neat PAA,^[^ ^35^ ^]^ primarily because the good adhesive properties of xPUU1k enable homogeneous coverage over the Si particles, thereby facilitating the formation of thin and compact SEI layer with much less pulverization or reconstruction.^[^ ^36^ ^]^ As further corroborated by the SEM images in Figure 4g, the overall spherical morphologies of the Si particles were preserved well in the xPUU1k electrode after 270 cycles. In contrast, the PAA electrode showed aggregated Si particles with ill‐defined morphologies wrapped by thick SEI layers. As shown in Figure 4h, the xPUU1k cell exhibited superior rate capability over the PAA cell under various C‐rates, attributable to the fast charge transfer enabled by the compact SEI layer formation, leading to efficient electrochemical reaction kinetics, particularly under high current densities. Indeed, as further demonstrated by determining the lithium‐ion diffusivities by the Randles–Sevcik equation (Figure 4i; Figure S11, Supporting Information),^[^ ^37^ ^]^ the xPUU1k cell exhibited 1.12 times higher lithium‐ion diffusivity than the PAA cell in both anodic and cathodic reactions, demonstrating the efficient electrochemical kinetics by adopting the xPUU1k binder.^[^ ^35^ ^]^ Consequently, a high‐loading silicon anode with areal mass loading of 1.3 mg cm^−2^ prepared using the xPUU1k binder demonstrated areal capacity exceeding 1.7 mAh cm^−2^ for 200 cycles (capacity retention of 65%), further proving its outstanding binder performance (Figure S12, Supporting Information).

**Figure 5:**
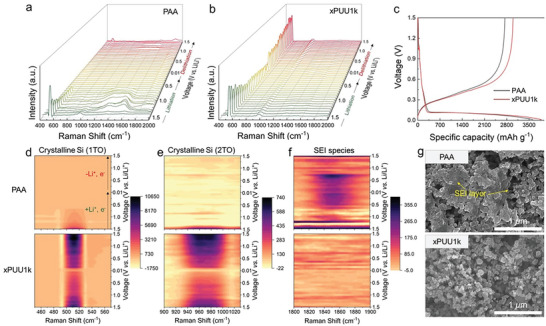
In‐situ operando Raman spectroscopic characterizations of the Si electrodes. Full‐range in‐situ Raman spectra of the a) PAA and b) xPUU1k cell during the first cycle of lithiation/delithiation under 0.2 C. c) Voltage‐capacity profiles of the PAA and xPUU1k cell during the in‐situ Raman spectroscopic measurement. Contour maps of the PAA (top) and xPUU1k (bottom) cell in the regime showing d) Si 1st order transverse optical (1TO) mode, e) Si 2nd order transverse optical (2TO) mode, and f) SEI species. g) Surface SEM images of the PAA (top) and xPUU1k (bottom) cell after the measurement.

In order to probe the real‐time structural changes in the Si electrodes, in‐situ operando Raman spectroscopy was performed during the initial lithiation/delithiation cycle. **Figure** **5**a,b shows the waterfall charts of the Raman spectra obtained from the PAA and xPUU1k cells. Figure 5c presents the corresponding voltage‐capacity profiles during cycling, where the xPUU1k cell delivers a higher specific capacity with a smaller overpotential than the PAA cell. As shown in Figure 5a,b, it is interesting to note that the PAA and xPUU1k cells exhibit completely different portraits of Raman spectra, particularly in the proximity of 520, 960, and 1850 cm^−1^, which correspond to the characteristic Raman signals of 1st order transverse optical (1TO) mode of crystalline Si, 2nd order TO (2TO) mode of crystalline Si, and SEI species, respectively.^[^ ^38^ ^]^ To better expose the peak intensity changes upon cycling, contour mapping of the Raman spectra was conducted as presented in Figure 5d–f. Notably, as shown in Figure 5d,e, the 1TO and 2TO signals originating from the crystalline Si detected from the PAA cell rapidly diminished at the early stage of the lithiation step.^[^ ^38^ ^]^ Given that Si lithiation occurs below 0.2 V, such a premature and complete disappearance of the peak is not necessarily ascribed from the Si amorphization but primarily associated with the formation of the thick SEI layer that blocks the Si signal, as previously reported.^[^ ^38^, ^39^ ^]^ In sharp contrast, both the 1TO and 2TO signals persist over cycling in the xPUU1k cell, indicative of the formation of a thin and compact SEI layer, where both signals become gradually weaker as the voltage approaches 0.1 V at which the Si crystal is lithiated, and then stronger again upon delithiation with increasing the applied voltage.^[^ ^39^ ^]^ Furthermore, as shown in Figure 5f, a signal at around 1850 cm^−1^ associated with the SEI layer species, as previously documented by Krause et al.,^[^ ^38^ ^]^ evolved almost simultaneously with the diminution of the crystalline Si signals from the PAA cell, while no appreciable change was detected from the xPUU1k cell. This observation also indicates that a relatively thin and uniform SEI layer is formed on the Si surface in the xPUU1k cell compared to the PAA cell, primarily enabled by the highly elastic and adhesive features of the xPUU1k binder, concurrently assisted by the retention of strong hydrogen bonding upon cycling. Indeed, as further verified by SEM analysis on the surface morphologies of the cycled Si electrodes after Raman spectroscopy (Figure 5g), Si particles in the PAA electrode were mostly covered by thick SEI layers, whereas the xPUU1k electrode exhibited nearly the same Si morphologies with the pristine state without being much passivated (Figure S13). These findings highlight that binder plays a significant role in the SEI layer formation in the Si electrode.

**Figure 6:**
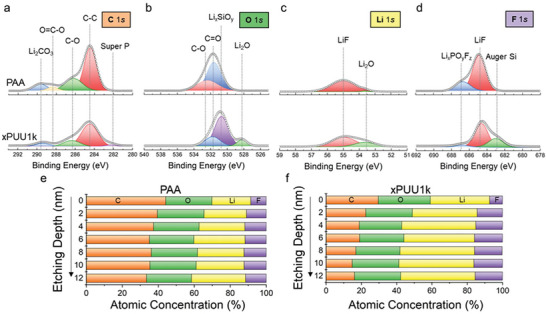
In‐depth XPS analysis on the SEI compositions of the Si electrodes. Representative deconvoluted a) C 1*s*, b) O 1*s*, c) Li 1*s*, and d) F 1*s* XPS spectra of the cycled PAA (top) and xPUU1k (bottom) electrode after 270 cycles under 0.5 C at 30 °C. In‐depth profiling of the atomic concentrations of e) PAA and f) xPUU1k electrode.

To identify the detailed chemical composition of SEI layers formed on the Si electrodes, in‐depth XPS profiling combined with ion‐sputtering was performed (**Figure** **6**). Note that both the PAA and xPUU1k electrodes showed nearly the same composition before cycling (Figure S14, Supporting Information). As shown in the representative deconvoluted C 1*s* spectra (Figure 6a), significantly larger amounts of carbon‐containing species, including Li_2_CO_3_, alkyl carbonate, and alkyl carboxylates, as the electrolyte decomposition products were detected from the PAA electrode compared to the xPUU1k electrode after 270 cycles.^[^ ^40^ ^]^ Furthermore, a signal at around 282 eV corresponding to the Super P was exclusively detected from the xPUU1k electrode, indicating the formation of a thin SEI layer that did not block the Super P signal.^[^ ^41^ ^]^ In the O 1*s* and Li 1*s* spectra (Figure 6b,c), the xPUU1k electrode exhibits prominent Li_x_SiO_y_ and Li_2_O peaks, which are typically formed in the vicinity of Si particles.^[^ ^40^, ^42^ ^]^ Given that those peaks were barely detectable from the PAA electrode, we speculate that a substantially thick SEI layer was formed on the Si surface in the PAA electrode. In addition, a significantly larger amount of Li_x_PO_y_F_z_ peak at about 687 eV was detected from the PAA electrode compared to the xPUU1k electrode (see Figure 6d) indicating that a larger amount of LiPF_6_ is decomposed in the PAA electrode than the xPUU1k electrode.^[^ ^35^ ^]^ Furthermore, the peak at 683 eV corresponding to the Auger Si only evolved from the xPUU1k electrode, not from the PAA electrode, which could be additional evidence of thin SEI layer formation.^[^ ^43^ ^]^ To further probe the spatial distribution of the SEI species, XPS depth profiling was conducted using Ar ion etching, as shown in Figure 6e,f. As is typically found in the previous reports, the C and O content continuously decreases with the etching depth from both the PAA and xPUU1k electrodes, primarily because organic species comprising carbon and oxygen exist mostly on the outer layer.^[^ ^44^ ^]^ Given that the fractions of the C and O components in the PAA electrode were considerably larger than those from the xPUU1k electrode, the PAA electrode should possess a thicker and inhomogeneous SEI layer compared to the xPUU1k electrode.^[^ ^40^ ^]^ Hence, all of these findings point to the powerful role that binders play in the Si electrodes, particularly in constructing a thin and uniform SEI layer that is desirable for achieving long‐lasting Si anodes.

## 3 Conclusion

We have demonstrated that interweaving the highly elastic PUU elastomer with the rigid PAA by cross‐linking leads to accessing a delicate balance between the distinct mechanical features, thereby simultaneously achieving mechanical robustness and elasticity derived from PAA and PUU, respectively. Systematic modulation, particularly in the length of hard/soft segments and the structure of the hard segment component of the xPUU system, essentially dictates various aspects of mechanical properties, wherein the xPUU1k exhibits the most elastic, tough, and stress‐relaxable features across the xPUU# series. Consequently, the highly elastic PUU1k chains anchored on the PAA backbone effectively enable stress dissipation through chain stretching during lithiation, while the structural integrity was retained well upon delithiation due to facile hydrogen bonding between the urea/urethane moieties and the silanol. Furthermore, the elastic and adhesive features of the xPUU1k also contribute to constructing a thin SEI layer on the Si surface upon cycling, as revealed by in‐situ operando Raman and XPS analyses. All the desirable mechanical properties of xPUU1k lead to superior cycling performance when used as a Si electrode binder, compared to the neat PAA binder. This study offers a fresh perspective on the rational design of Si binders, primarily focusing on the mechanical properties of polymer binders to access long‐lasting Si electrodes. We believe that the set of findings establishes a clear perspective to the open question of which mechanical properties of polymers are desirable to alleviate the failure mechanism of Si electrodes.

## 4 Experimental Section

### Materials

Poly(ethylene glycol) (PEG, average *M*_n_ ≈400, 1k, 2k, and 6k g mol^–1^), pyridine–2,6–dicarboxylic acid, thionyl chloride, *m*–phenylenediamine, 4,4–methylenedianiline, hexamethylene diisocyanate (HMDI), di–*tert*–butyl dicarbonate, triethylamine (TEA), 4–(dimethylamino)pyridine (DMAP), poly(acrylic acid) (PAA, average *M*_v_ ≈450,000 g mol^–1^), *N*‐methyl‐2‐pyrrolidone (NMP), and dibutyltin dilaurate (DBTL) were purchased from Aldrich. Silicon nanopowder (Si, 99%, 30–50 nm) was purchased from NanoAmor. 1 m lithium hexafluorophosphate (LiPF_6_) in a mixture of ethylene carbonate (EC) and diethyl carbonate (DEC) (1:1 vol%) with 10 wt.% of fluoroethylene carbonate (FEC) was purchased from Welcos Co., Ltd (Korea). Trilayer polypropylene/polyethylene/polypropylene (PP/PE/PP, Celgard2320, thickness: 25 µm) was used as a separator for electrochemical analyses. All other chemicals and solvents were purchased from reliable commercial sources and used as received.

### Synthesis of Boc‐Protected *N* ^2^, *N* ^6^–bis(3–aminophenyl)pyridine–2,6–dicarboxamide

Pyridine–2,6–dicarbonyl dichloride and tert‐butoxycarbonyl (mono‐Boc) protected m–phenylenediamine were synthesized as described in the literature.^[^ ^23^, ^45^ ^]^ Mono‐Boc‐protected m–phenylenediamine (25.89 g, 124.3 mmol), TEA (13.84 g, 136.7 mmol), and DMAP (1.519 g, 12.43 mmol) were dissolved in anhydrous methylene chloride (750.0 mL). A solution of pyridine–2,6–dicarbonyl dichloride (12.68 g, 62.15 mmol) was added slowly, and the resulting mixture was stirred at room temperature for 16 h. After removing the solvent under a dynamic vacuum, the crude product was filtered and washed using methanol. After being dried under a dynamic vacuum, a white solid was obtained with a 71% yield. ^1^H NMR (400 MHz, DMSO‐*d*_6_, *δ*): 11.02 (NH‐CO, 2H), 9.48 (NH‐COO, 2H), 8.38 (pyridine, 2H), 8.32 (pyridine, 1H), 8.14 (Ar‐H, 2H), 7.43 (Ar‐H, 2H), 7.29 (Ar‐H, 2H), 7.20 (Ar‐H, 2H), 1.49 (‐CH_3_, 18H).

### Synthesis of *N* ^2^, *N* ^6^–bis(3–aminophenyl)pyridine–2,6–dicarboxamide (bAPC)

The Boc‐protected *N* ^2^, *N* ^6^–bis(3–aminophenyl)pyridine–2,6–dicarboxamide was dissolved in a methylene chloride/trifluoroacetic acid (3:1 vol%) mixture and stirred at room temperature. After 3 h, an aqueous 10 wt.% sodium hydroxide solution was added to the solution until a white precipitate was formed. The resulting precipitate was filtered, washed with distilled water three times, and dried under a dynamic vacuum. ^1^H NMR (400 MHz, DMSO‐*d*_6_, *δ*): 10.79 (N*H*‐CO, 2H), 8.35 (pyridine, 2H), 8.29 (pyridine, 1H), 7.20 (Ar‐*H*, 2H), 7.05 (Ar‐*H*, 4H), 6.41 (Ar‐*H*, 2H), 5.17 (N*H*_2_, 4H).

### Synthesis of poly(urea‐urethane)# (PUU#)

Poly(urea‐urethane) was referred to as PUU#, where # indicates the molecular weight of PEG. The following representative synthetic procedure was for PUU1k. PEG1k (2.00 g, 2.00 mmol) and bAPC (2.00 g, 5.76 mmol) were dissolved in anhydrous DMF (21.3 mL) at 70 °C, and HMDI (1.30 g, 7.76 mmol) was added to the solution, followed by addition of 25.3 µL of DBTL. After stirring for 48 h at 70 °C, HMDI (1.30 g, 7.76 mmol) was added to the mixture to prepare isocyanate‐terminated PUU, and the reaction further proceeded for 3 h. The reaction mixture was cooled down to 40 °C, and the two‐equivalent glycidol (1.15 g, 15.5 mmol) was added to the solution with 12.7 µL of DBTL. The temperature was increased to 70 °C, and the reaction further proceeded for 3 h. The crude product was precipitated in diethyl ether three times, and the precipitate was filtered and dried under reduced pressure to obtain PUU1k. ^1^H NMR (400 MHz, DMSO‐*d*_6_, *δ*) of PUU1k: 10.96 (N*H*‐CO in bAPC, 2H), 8.54 (N*H*‐CO‐NH, 2H), 8.36 (pyridine, 2H), 8.28 (pyridine, 1H), 7.98 (Ar‐*H*, 2H), 7.15‐7.40 (Ar‐*H*, 6H, N*H*‐COO, 2H), 6.13 (NH‐CO‐N*H*, 2H), 5.17 (N*H*_2_, 4H), 4.02 (PEG, 4H), 3.50 (PEG, 85.3H), 3.09 (HMDI, 2H), 2.96 (HMDI, 2H), 1.13‐1.55 (HMDI, 8H).

### Preparation of Si Electrodes

Si nanopowder (60 wt%) was dispersed in *N*‐methyl‐2‐pyrrolidone (NMP) with Super P (20 wt.%) and binder (20 wt.%) using a Thinky mixer. The resultant slurry was uniformly cast onto a copper foil using a doctor blade, and the residual NMP solvent was removed under a dynamic vacuum for 10 h at 120 °C. The mass loading of the active materials in the Si electrode was in the range of ≈0.6–0.7 mg cm^−2^.

### Electrochemical Characterizations

Charge/discharge cycling test of Li/Celgard2320/Si cell was conducted at a cut‐off voltage range of 0.01–1.5 V (vs Li/Li^+^) at 30 °C, where 1 C corresponds to a current density of 3000 mA g^−1^. 1 M LiPF_6_ in EC:DEC (1:1 vol%) with 10 wt.% FEC was used as a liquid electrolyte, and all the cell components were assembled in an Argon‐filled glove box (O_2_ < 0.1 ppm, H_2_O < 0.1 ppm). Electrochemical impedance spectroscopy (EIS) was performed using a VMP3 multi‐channel potentiostat (Biologic, France) in a frequency range from 0.1 to 100 MHz with 10 mV of amplitude at room temperature. In‐situ Raman spectroscopy was performed using a confocal micro‐Raman spectrometer (Renishaw, In Via Raman Microscope) with 532 nm Nd:Yag laser, and the scattered light was collected using an ×50 optical lens. For the in‐situ measurement, a Si electrode slurry was cast onto a copper mesh current collector and assembled in a 2032‐coin cell with a hand‐made case containing a quartz glass window in the center. Li metal was used as a counter electrode and the cell was cycled under 0.2 C during the in‐situ Raman spectroscopic measurement. Cyclic voltammetry (CV) was performed using a VMP3 multi‐channel potentiostat (Biologic, France) in a voltage range of 0–1.5 V (vs Li/Li^+^) at a scan rate of 0.05–0.2 mV s^−1^.

### Instrumentation and Characterization Techniques

^1^H NMR spectra were recorded on an Ascend 400 spectrometer (400 MHz) using CDCl_3_ and DMSO‐*d*_6_ (Cambridge Isotope Laboratories) as solvents at room temperature, with tetramethylsilane (TMS) as a reference. Inverse‐gated ^13^C NMR spectra were recorded on an Avance600 spectrometer (600 MHz) using DMSO‐*d*_6_ (Cambridge Isotope Laboratories) as a solvent at room temperature, with TMS as a reference. Molecular weights (*M*_n_ and *M*_w_) and polydispersity index (PDI) were determined by gel permeation chromatography (GPC) using the Ultimate 3000 HPLC system (Thermo Fisher Scientific Inc., USA). HPLC grade DMF (J. T. Baker) was used as an eluent. The Fourier‐transform infrared (FT‐IR) spectra were recorded in the absorption mode on a Nicolet 6700 spectrophotometer with a resolution of 4 cm^–1^ in the vibrational frequency range from 400 to 4000 cm^−1^. The tensile tests were conducted using the Lloyd‐LS1 universal tensile machine (Lloyd, UK) with a cross‐head speed of 5 mm min^−1^. The polymer samples were cut into dog‐bone‐shaped specimens using the ASTM standard D638‐M (type V specimens), and at least five measurements were performed for each sample to determine the reliable average values. The 180° peeling tests were carried out using the Lloyd‐LS1 with a cross‐head speed of 10 mm min^−1^. The 3 m double‐sided tape was attached to the electrode samples with a dimension of 1.2×2.0 cm^2^ using a rubber roller (diameter: 95 mm, width: 45 mm, weight: 2.0 kg) to ensure consistent pressure through the experiments. The adhesive force was recorded upon being peeled at a 180° angle. Nano‐indentation test was performed using a TI950 Triboindenter (Hysitron, USA) with a displacement control of 500 nm. The creep‐recovery tests were carried out using the Discovery Hybrid Rheometer (DHR‐2, TA Instruments, USA), equipped with an 8 mm parallel plate geometry within the linear viscoelastic regime. The samples were loaded with a constant stress of 20 kPa for 120 s, followed by 130 s of recovery by removing the stress. Dynamic mechanical analysis was conducted using the SDTA861e (Mettler Toledo) equipped with a tensile head. Rectangular specimens with a dimension of 25×5.0×0.2 mm^3^ were deformed by a force amplitude of 10 N and displacement amplitude of 10 µm in the 0.1–100 Hz frequency range. To establish a time‐temperature superposition (TTS) master curve, data was collected at intervals of 10 and 20 °C in the 30–70 and 70–150 °C ranges, respectively. X‐ray photoelectron spectroscopy (XPS) was performed using a PHI 5000 VersaProbe (ULVAC PHI, Japan) with a monochromatized Al Kα as a radiation source. A survey spectrum was collected over a range of 0–1400 eV, followed by high‐resolution narrow scans of C1s, O1s, F1s, and Li1s regions. Depth profiles were collected using an Ar^+^ sputter source with a sputter rate of 0.5 nm s^−1^. Scanning electron microscopy (SEM) was performed on a Regulus 8230–Oxford EDS (Hitachi Inc., Tokyo, Japan) at an accelerating voltage of 10 kV. SEM specimens were prepared by washing with dimethyl carbonate (DMC) followed by vacuum drying at room temperature in an Argon‐filled glove box.

## Conflict of Interest

The authors declare no conflict of interest.

## Supporting information

Supporting Information

## Data Availability

The data that support the findings of this study are available from the corresponding author upon reasonable request.
