# Supplementary material for: Interweaving Elastic and Hydrogen Bond‐Forming Polymers into Highly Tough and Stress‐Relaxable Binders for High‐Performance Silicon Anode in Lithium‐Ion Batteries
Source: Adv Sci (Weinh). 2023 Sep 26;10(31):2302027. doi: 10.1002/advs.202302027 (PMC10625081; doi:10.1002/advs.202302027)
Supplement: Supplementary file 1 — Supporting Information [file ADVS-10-2302027-s001.pdf]

ADVANCED SCIENCE
Open Access

Supporting Information

for *Adv. Sci.*, DOI 10.1002/advs.202302027

Interweaving Elastic and Hydrogen Bond-Forming Polymers into Highly Tough and Stress-Relaxable Binders for High-Performance Silicon Anode in Lithium-Ion Batteries

Daun Jeong, Jinsol Yook, Da-Sol Kwon, Jimin Shim and Jong-Chan Lee**

Supporting Information

Interweaving Elastic and Hydrogen Bond-Forming Polymers into Highly Tough and Stress-Relaxable Binders for High-Performance Silicon Anode in Lithium-Ion Batteries

Daun Jeong, Jinsol Yook, Da-Sol Kwon, Jimin Shim, * *and Jong-Chan Lee* *

D. Jeong, D.-S. Kwon, J. Shim
Energy Storage Research Center
Korea Institute of Science and Technology (KIST)
14 Gil 5 Hwarang-ro, Seongbuk-gu, Seoul 02792, Republic of Korea
E-mail: jshim@kist.re.kr

J. Yook, J.-C. Lee
School of Chemical and Biological Engineering and Institute of Chemical Processes
Seoul National University
1, Gwanak-ro, Gwanak-gu, Seoul 08826, Republic of Korea
E-mail: jongchan@snu.ac.kr

D.-S. Kwon
Department of Chemical and Biological Engineering
Korea University
145, Anam-ro, Seongbuk-gu, Seoul 02841, Republic of Korea

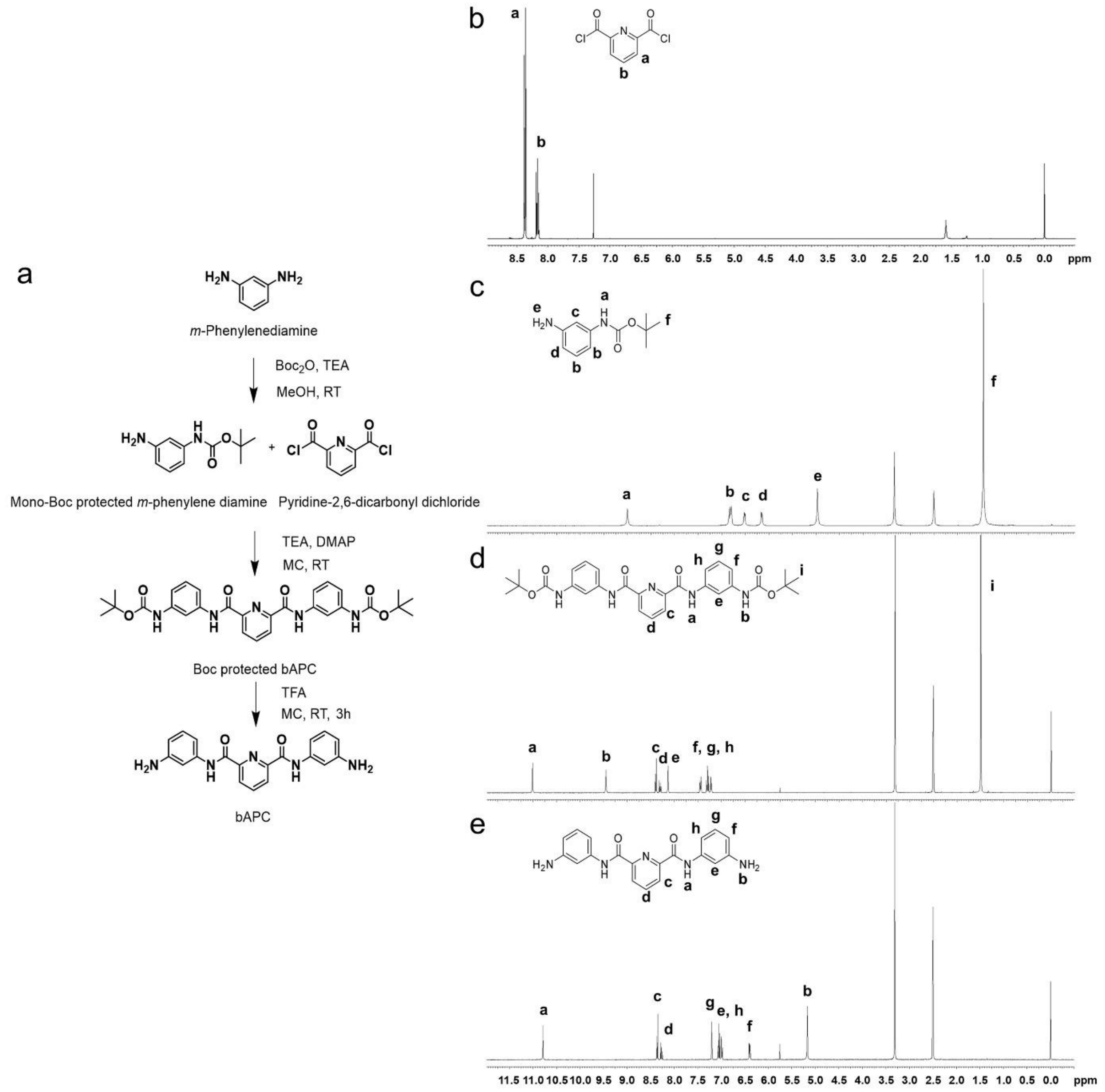

Figure S1. a) Synthetic route to bAPC. ^{1}H NMR spectrum of b) pyridine–2,6–dicarbonyl dichloride, c) mono-Boc-protected *m*–phenylenediamine, d) Boc-protected bAPC, and e) bAPC.

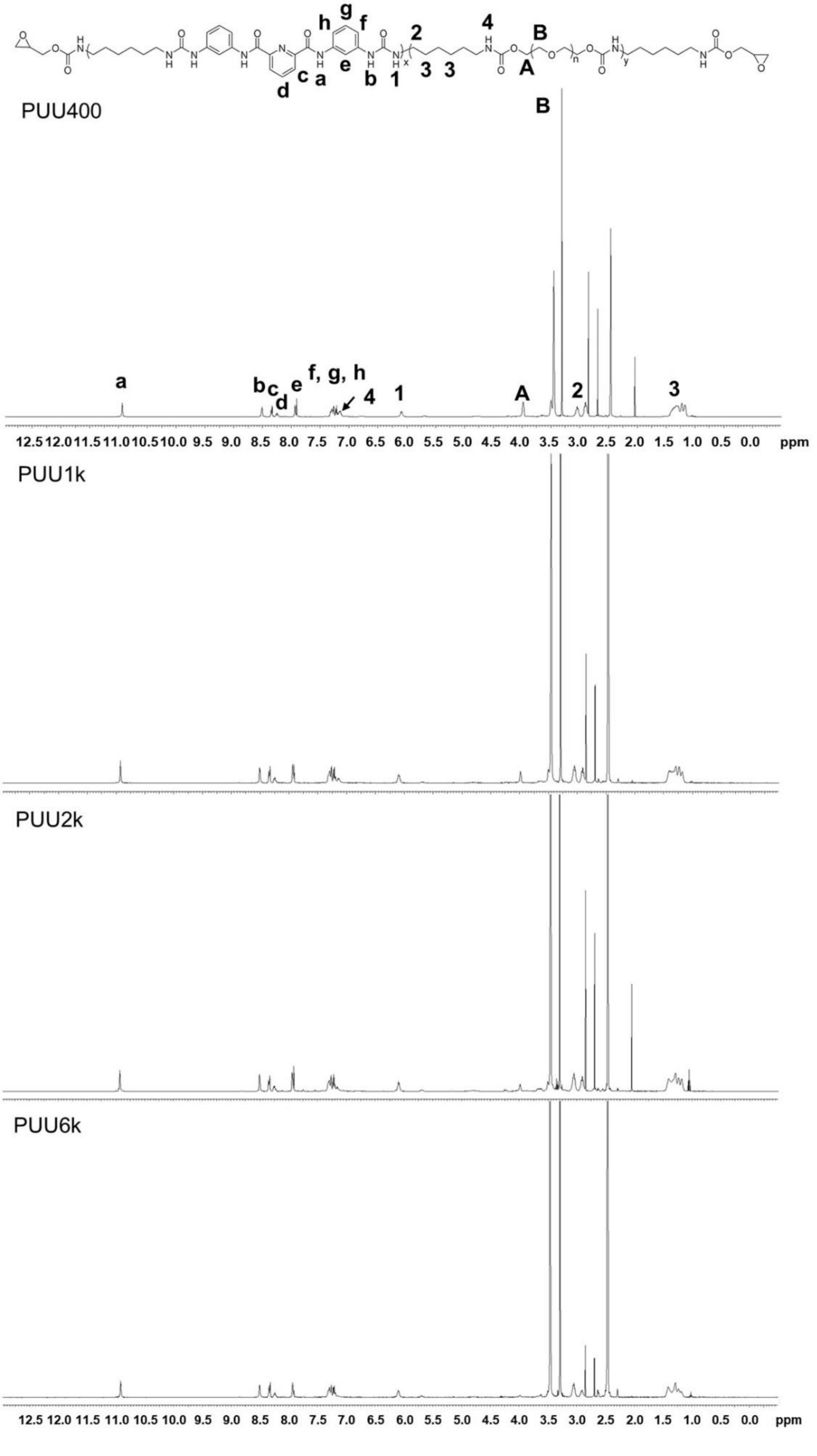

Figure S2. ^{1}H NMR spectra of PUU#s, where # represents the molecular weights of PEG.

Table S1. Molecular characteristics of PUU#s.

Samples	A weight ratio of PEG to bAPC[a]	M_n (g mol^{-1})[b]	PDI[b]
PUU400	1.17	37600	2.51
PUU1k	1.19	37400	2.09
PUU2k	1.24	39400	2.20
PUU6k	1.33	39600	2.49

a)Determined by ^{1}H NMR spectroscopy.

b)Determined by GPC analysis using DMF as the eluent.

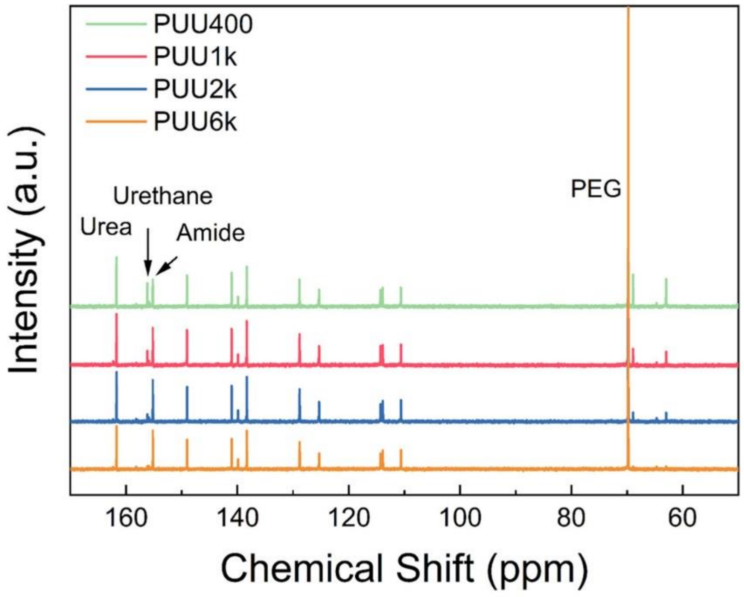

Figure S3. Inverse-gated ^{13}C NMR spectra of PUU#s.

Table S2. The carbon number of urea, urethane, amide, and PEG in PUU#s.

Samples	Urea	Urethane	Amide	PEG	M_n (g mol^{-1})[a]	
					Hard segment	Soft segment
PUU400	2.44	2.18	2.54	18.2	630	570
PUU1k	5.43	1.94	5.15	45.5	1400	1170
PUU2k	9.22	2.12	9.93	90.9	2380	2170
PUU6k	27.6	2.73	26.5	273	7130	6170

[a)] Determined by inverse-gated ^{13}C NMR spectra in **Figure S3**.

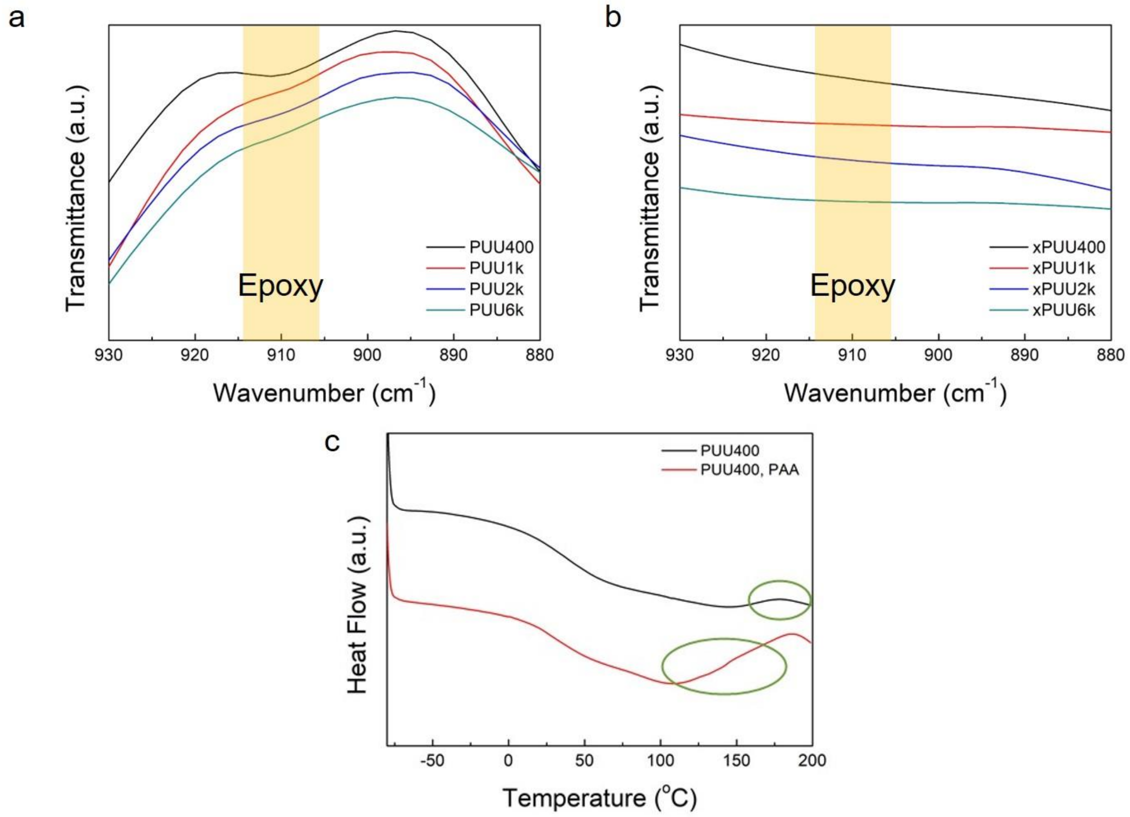

Figure S4. FT-IR spectra of the series of a) PUU#s and b) xPUU#s. c) Representative DSC thermograms of PUU400 and PUU400/PAA mixture (90/10 wt%).

As shown in the FT-IR spectra, the IR bands corresponding to the epoxy moieties at around 910 cm^{-1} (**Figure S4a**) disappear upon being heated at 120 °C (**Figure S4b**), indicating that the telechelic epoxy groups of PUU#s react with the carboxylic acid moieties of PAA. We speculate that carboxylic acid groups of PAA preferably react with epoxy groups rather than the urethane and urea moieties of PUU#s, given that the cross-linking temperature, 120 °C, is lower than the endothermic peak (initiating at 150 °C) of PUU#, as presented in the DSC thermograms (**Figure S4c**).

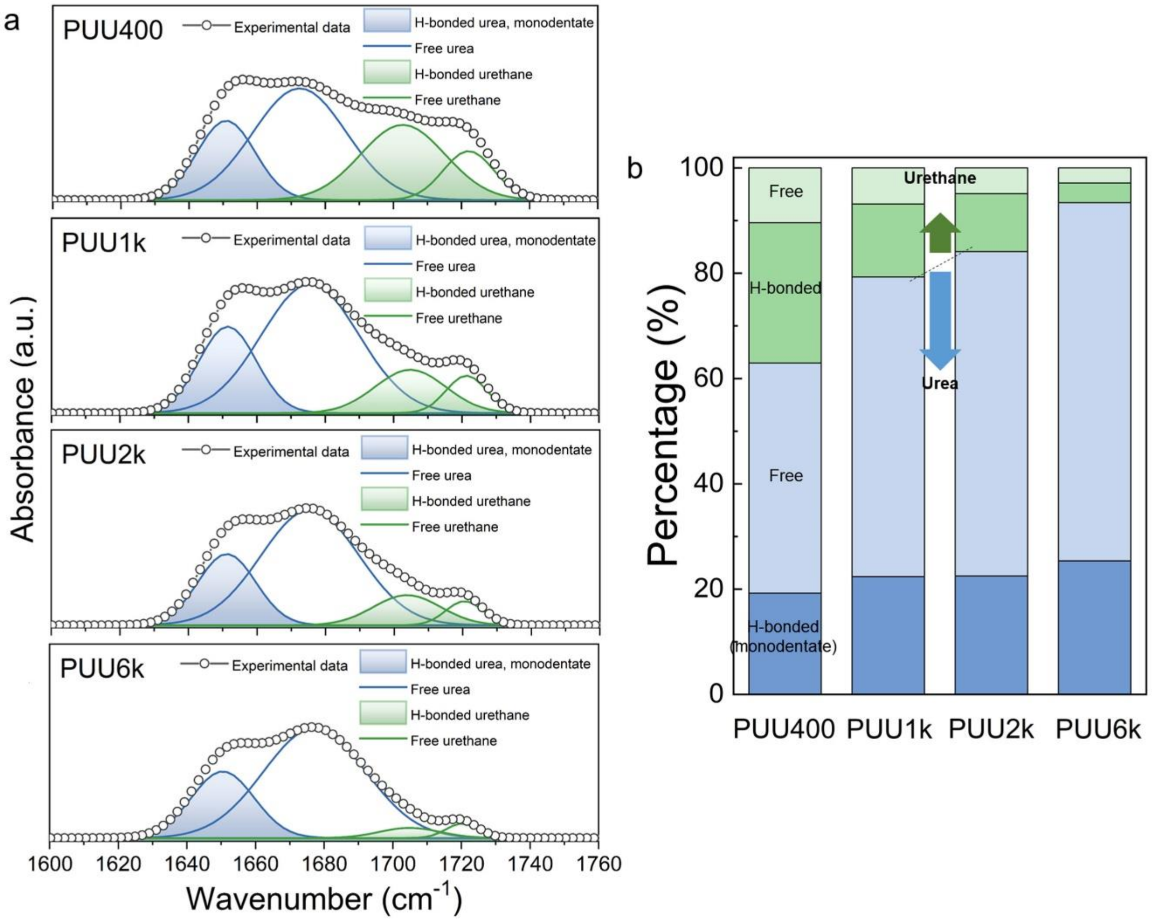

Figure S5. a) Deconvoluted FT-IR spectra of PUU#s displaying hydrogen (H)-bonded and free C=O stretching band. b) Percentages of H-bonded and free urea/urethane moieties of PUU#s.

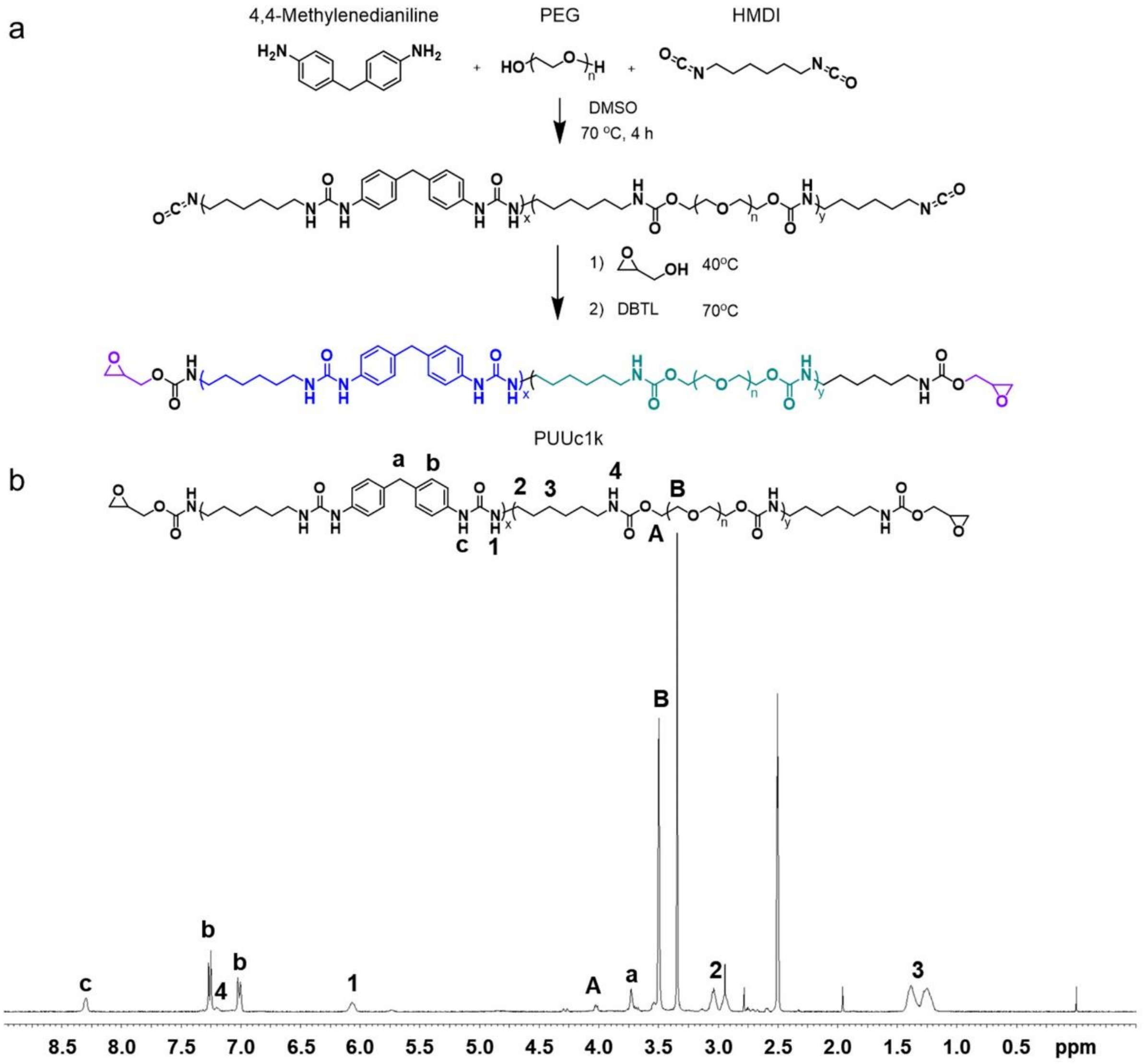

Figure S6. a) Synthetic route to PUUc1k. b) ^{1}H NMR spectrum of PUUc1k.

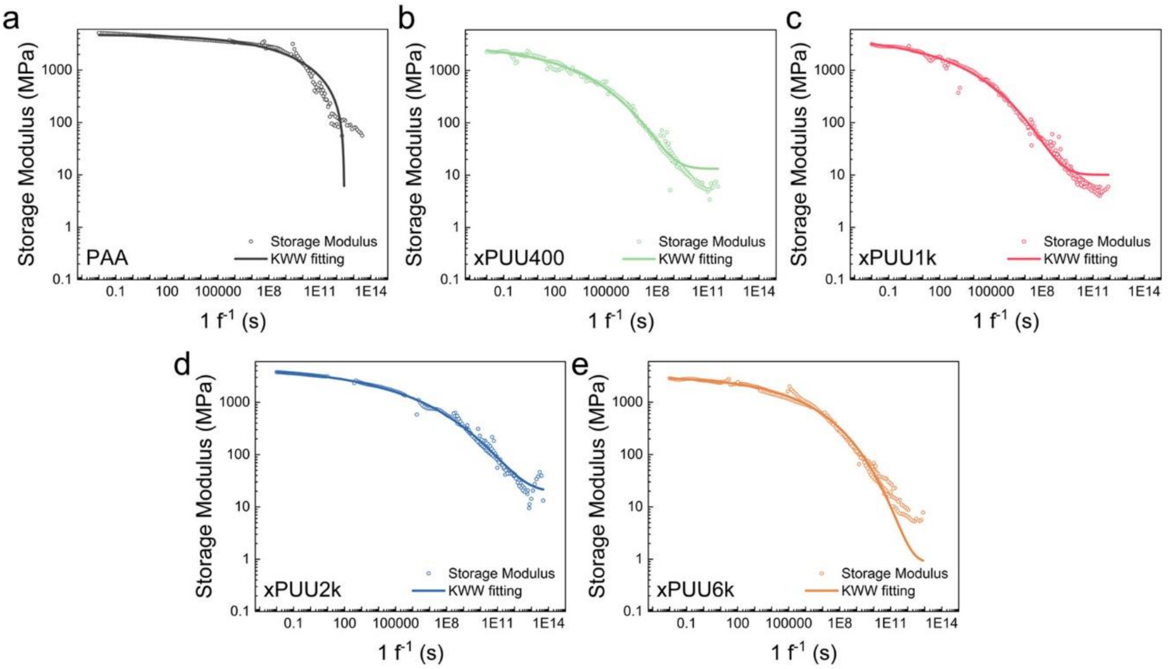

Figure S7. The experimental time-temperature superposition (TTS) master curves of storage modulus in the temperature range of 30–150 °C at T_{ref} of 30 °C (open symbols) and the corresponding Kohlrausch-Williams-Watts (KWW) fitting curves (full lines) of a) PAA, b) xPUU400, c) xPUU1k, d) xPUU2k, and e) xPUU6k.

Table S3. Relaxation time (τ_{KWW}) and free volume (f_0) of PAA and xPUU#s at 30 °C.

	τ_{KWW} (s)[a)]	f_0 (%)[b)]
PAA	1.01×10^{10}	1.15
xPUU400	3.75×10^{2}	2.71
xPUU1k	1.37×10^{2}	2.58
xPUU2k	3.32×10^{4}	2.52
xPUU6k	2.20×10^{4}	2.05

[a)]The Relaxation time (τ_{KWW}) is determined by Kohlrausch-Williams-Watts (KWW) function:

$$E(\tau) = E_\infty + (E_0 - E_\infty)exp\left[-\left(\frac{\tau}{\tau_0}\right)^\beta\right]$$

, where E is the storage modulus, E_0 is the instantaneous modulus, E_∞ is the relaxed modulus (E at $\tau = \infty$), τ is the reduced time variable ($\tau = t\,\alpha_T^{-1}$), τ_0 is the relaxation time at the reference temperature, and β is the distribution parameter.

[b)]The free volume (f_0) is determined by Williams-Landel-Ferry (WLF) equation,

$$\log(\alpha_T) = \frac{-C_1(T - T_{ref})}{C_2 + (T - T_{ref})}$$

,where α_T is the horizontal shift factor at certain temperature, T_{ref} is the reference temperature (30 °C), and C_1 and C_2 are the two material parameters to be identified.

The free volume (f_0) at the reference temperature can be determined by the following equation:

$$C_1 = \frac{B}{2.303 \times f_0}$$

, where *B* is the numerical constant, generally set as unity.

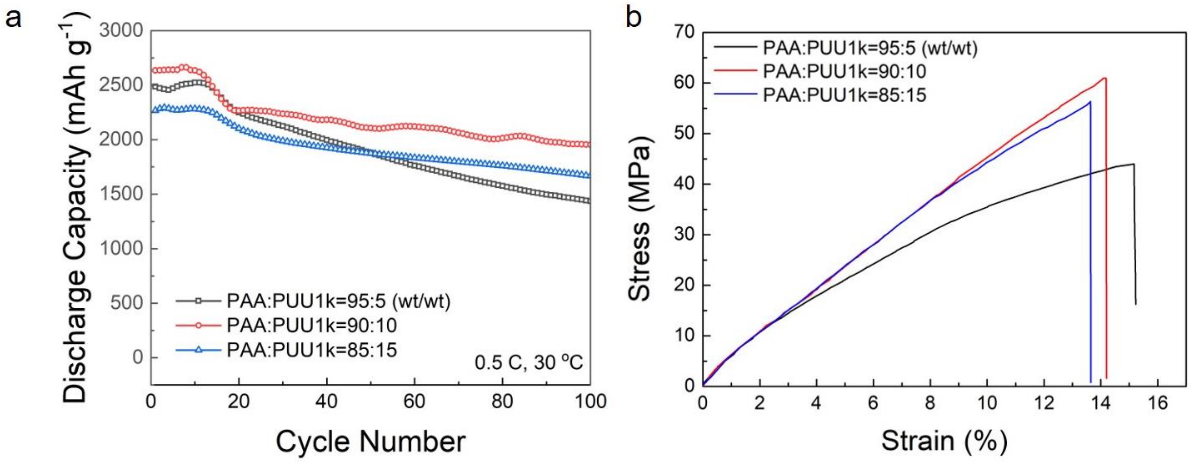

Figure S8. a) Cycling performance of Li/Celgard®2320/Si cells cycled under 0.5 C at 30 °C and b) stress-strain curves of xPUU1k, where the PUU1k content in the xPUU1k was varied as 5, 10, and 15 wt%.

As shown in **Figure S8a**, the xPUU1k binder system containing 10 wt% of PUU1k delivers higher specific capacities with greater capacity retention than the other compositions including 5 and 15 wt% of PUU1k. This is presumably because the xPUU1k with 10 wt% of PUU1k shows the highest toughness among the series as shown in **Figure S8b**.

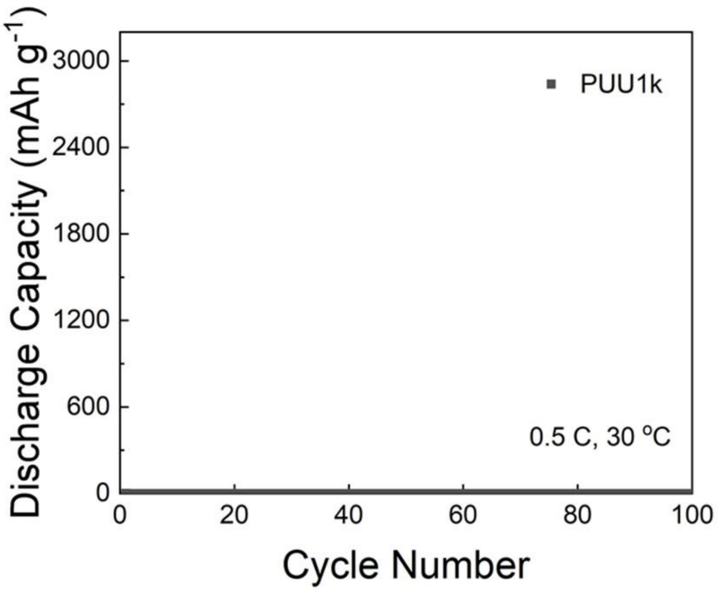

Figure S9. Cycling performance of Li/Celgard®2320/Si cell with PUU1k binder cycled under 0.5 C at 30 °C.

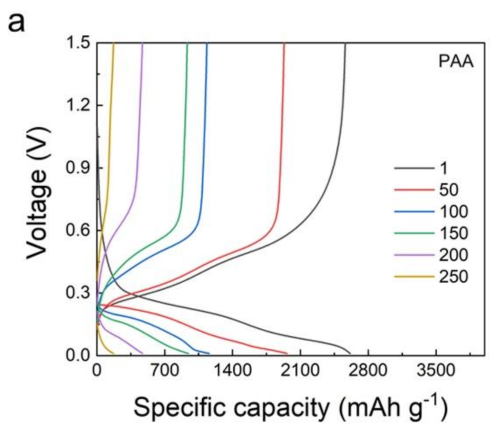

Figure S10. Voltage-capacity profiles of Li/Celgard®2320/Si cells with a) PAA and b) xPUU1k binder cycled under 0.5 C at 30 ℃.

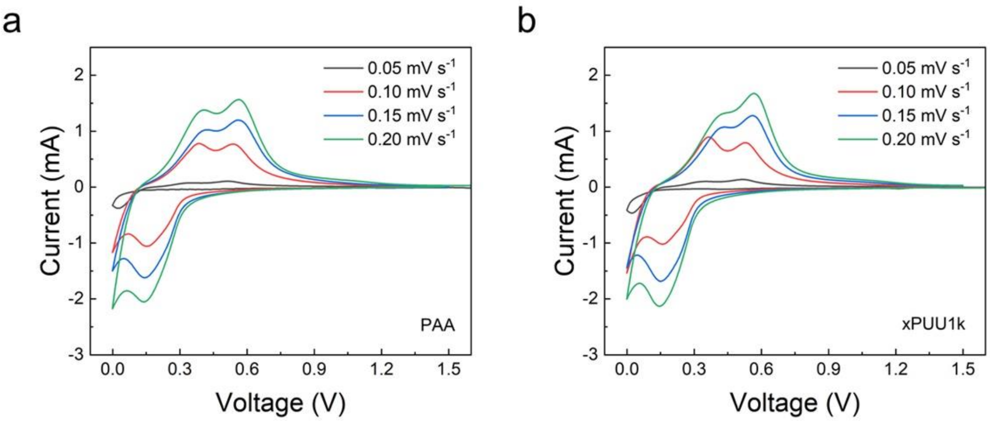

Figure S11. Cyclic voltammetry (CV) curves of Li/Celgard®2320/Si cells at different scanning rates with a) PAA and b) xPUU1k binder.

Lithium-ion diffusivity was determined by the Randles–Sevcik equation given by

$$I_p = (2.69*105) n^{3/2} C_{Li^+} A v^{1/2} D_{Li^+}{}^{1/2}$$

, where I_p is the peak current, n is the number of charge-transfer, A is the electrode area, C_{Li^+} is the concentration of lithium ion (Li^+) in the electrolyte, v is the potential scanning rate, and D_{Li^+} is the Li^+ diffusivity. The D_{Li^+} values are determined by using a slope of the linear plot of peak current (I_p) versus square root of potential scanning rate ($v^{0.5}$) from the CV curves at various scanning rates.[1]

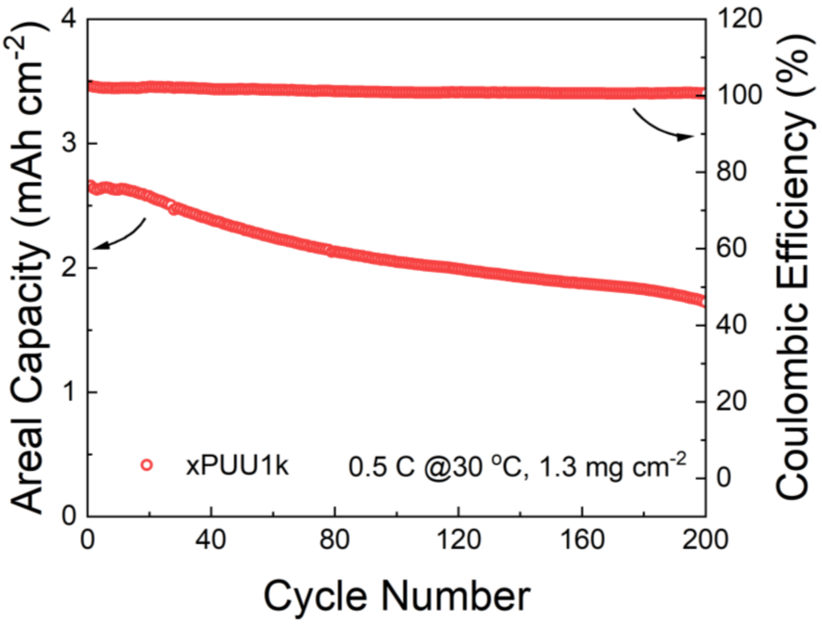

Figure S12. Cycling performance of high-loading Si anode prepared with the xPUU1k binder (areal mass loading: 1.3 mg cm^{-2}).

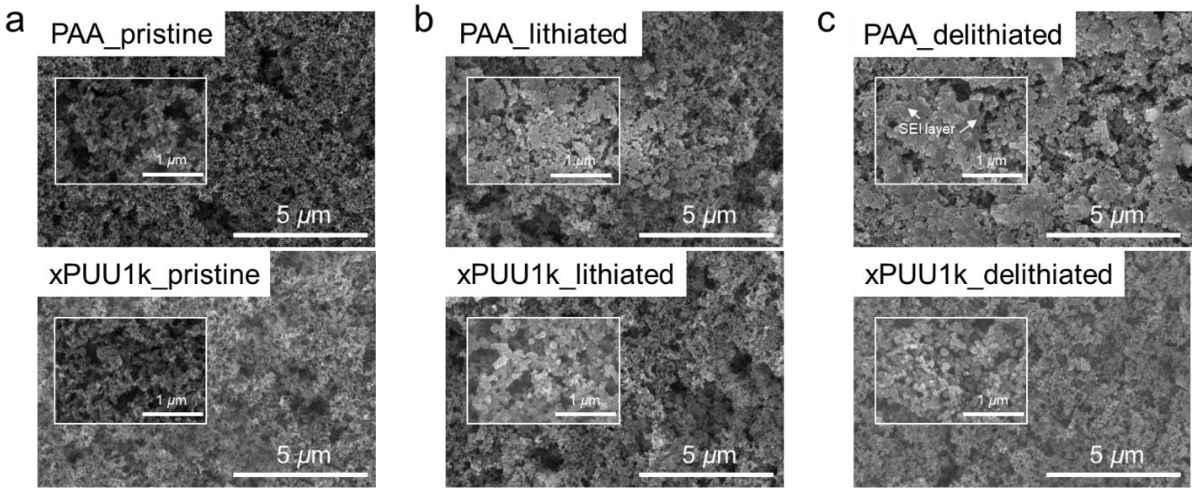

Figure S13. Surface SEM images of the a) pristine, b) lithiated, and c) delithiated PAA (top) and xPUU1k (bottom) electrodes cycled at 0.2 C.

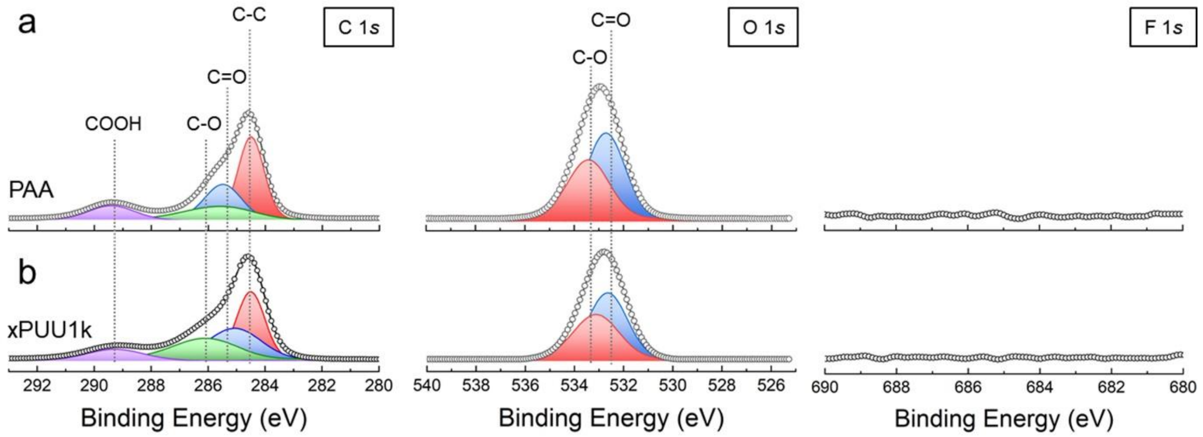

Figure S14. Surface C 1*s*, O 1*s*, and F 1*s* XPS profiles of the a) PAA and b) xPUU1k electrode before cycling.

Reference for Supporting Information

[1] L. Hu, M. Jin, Z. Zhang, H. Chen, F. B. Ajdari, J. Song, *Adv. Funct. Mater.* **2022**, *32*, 2111560.
